# Discovery of significant porcine SNPs for swine breed identification by a hybrid of information gain, genetic algorithm, and frequency feature selection technique

**DOI:** 10.1186/s12859-020-3471-4

**Published:** 2020-05-26

**Authors:** Kitsuchart Pasupa, Wanthanee Rathasamuth, Sissades Tongsima

**Affiliations:** 1 Faculty of Information Technology, King Mongkut’s Institute of Technology Ladkrabang, Bangkok, 10520 Thailand; 2 National Biobank of Thailand, National Science and Technology Development Agency, Khong Luang, 12120 Thailand

**Keywords:** Single nucleotide polymorphisms, Feature selection, Information gain, Genetic algorithm, Support vector machine

## Abstract

**Background:**

The number of porcine Single Nucleotide Polymorphisms (SNPs) used in genetic association studies is very large, suitable for statistical testing. However, in breed classification problem, one needs to have a much smaller porcine-classifying SNPs (PCSNPs) set that could accurately classify pigs into different breeds. This study attempted to find such PCSNPs by using several combinations of feature selection and classification methods. We experimented with different combinations of feature selection methods including information gain, conventional as well as modified genetic algorithms, and our developed frequency feature selection method in combination with a common classification method, Support Vector Machine, to evaluate the method’s performance. Experiments were conducted on a comprehensive data set containing SNPs from native pigs from America, Europe, Africa, and Asia including Chinese breeds, Vietnamese breeds, and hybrid breeds from Thailand.

**Results:**

The best combination of feature selection methods—information gain, modified genetic algorithm, and frequency feature selection hybrid—was able to reduce the number of possible PCSNPs to only 1.62% (164 PCSNPs) of the total number of SNPs (10,210 SNPs) while maintaining a high classification accuracy (95.12%). Moreover, the near-identical performance of this PCSNPs set to those of bigger data sets as well as even the entire data set. Moreover, most PCSNPs were well-matched to a set of 94 genes in the PANTHER pathway, conforming to a suggestion by the Porcine Genomic Sequencing Initiative.

**Conclusions:**

The best hybrid method truly provided a sufficiently small number of porcine SNPs that accurately classified swine breeds.

## Background

Purebred pigs are commercially important and many pig breeders request purebred pigs in their cross-breeding programs. Cross-breeding helps breeders discover new breeds with desirable traits, e.g., disease resistance and heat tolerance. Swine genetic diversity stems from genetic differences. The most prevalent differences are in the form of variation at the level of a nucleotide, termed single nucleotide polymorphism (SNP). A single nucleotide or base (adenine, guanine, cytosine, or thymine) substitution can cause changes at the protein level which results in changes of phenotypes. An informative SNP profile, a collection of porcine-classifying SNPs (PCSNPs) collected from a pig, can be used to represent a given phenotype.

Porcine SNPs can be used to classify pigs into different breeds. However, since there could be ten of thousand SNPs representing each pig, it may not be so practical (and costly) to use this large SNP collection as a molecular pig classification testing kit. Therefore, the original number of SNPs should be reduced by means of feature selection, i.e., a small number of SNPs that carry a statistical power to achieve good classification results. Moreover, selection of a small number of the most significant features for classification is very important because even though gene and SNP matching procedure is adequately efficient, the validation procedure for each match is extremely costly and so least significant SNPs are preferably not included. With the advent of AI and machine learning era, we can adopt well established algorithms to efficiently filter SNPs (feature selection). This study focused on bringing together a feature selection technique in combination with a classification technique in machine learning to apply to SNP selection and classification of swine breeds. The most popular classification techniques at the present time are Bayesian classifiers, nearest neighbor, neural networks, and support vector machines (SVM) [1]. They have been applied successfully on various types of data such as SNPs, proteomics, genomics, and microarray, all of which have a large number of features (called dimensions hereafter). These high dimensions affect the efficiency of classification techniques because some features are not necessary for the construction of an accurate classification model. Another issue is that, typically, the number of samples to be classified is very small, so there is a strong tendency that an overfitting issue may occur. An overfitting is a situation in which a classifier can model the training data too well (including noise in the data) but not others because the trained model is not generalized enough for other inputs.

Feature selection plays a crucial role in machine learning. Its importance is explained in [2], a review paper on the feature selection techniques for classification tasks. Kwak and Choi present an efficient feature selector that reduces computational time and provides accurate classification results [3]. The feature selection techniques commonly used in bioinformatics were reported in [4], especially pertaining to microarray. This study considered these techniques to be of three types–filter, wrapper, and embedded methods. A filter feature selection method applies a statistical measure to assign a score to each feature. The features are ranked by the score and either selected to be kept or removed from the data set. The features that are assigned a high score will be selected to be used in the further classification step. The advantages of this method are that it can be applied to a data set that has a large number of features in a simple and efficient way that is independent from the machine learning algorithms. As well as that it involves a lower risk of an overfitting issue which sets it apart from a wrapper method that entails a high risk of this issue. A disadvantage of this method is that it produces a feature set that is not tuned to a specific type of predictive model, so a filter method may fail to find the best subset of features for a particular predictive model in many occasions. A wrapper method, on the other hand, is dependent on machine learning algorithms. The final selected features are the features that provide the best result as the machine learning algorithms operate on a variety of subsets of features suggested by a search algorithm such as genetic algorithm (GA) and sequential search. Even though a wrapper method uses a long computational time from having to perform a lot of procedural steps, it often provides the best subset of features. The last type of methods, embedded method, is a feature selection method that is embedded in the machine learning algorithm which, in this sense, is similar to a wrapper method that is dependent on the machine learning algorithms, but it uses a shorter processing time.

Filter methods that have been widely used for bioinformatics tasks are such as *χ*^2^, *i*-test, gain ratio, Euclidean distance, correlation-based feature selection, and Markov blanket [4]. In [5], other filter methods than the ones reported in [4] are presented for applying on gene expression microarray data set. These methods are rank product, fold-change ratio, modified *t*-test, and information gain (IG). Thamwiwatthana, Pasupa, and Tongsima presented a technique to reduce the number of beta-thalassaemia SNPs of Thai population [6]. That study used several filters, embedded methods and classifiers as well as their combinations. The best combination was *χ*^2^+SVM that selected a small number of PCSNPs that can classify severe or mild cases of beta-thalassaemia. Besides filter methods, several wrapper methods have also been widely used. In two review papers [4, 7], several widely used wrapper methods are mentioned such as sequential search, simulated annealing, and nature-inspired algorithms. Methods in the nature-inspired group are such as binary particle swarm optimization, GA [8, 9], binary flower pollination [10], and binary cuckoo search [11]. The wrapper method of our interest was GA. Lastly, there have been extensive researches on embedded methods such as sparsity control by using *l*_*q*_-norm [12], Jeffrey’s Hyperprior [13], canonical variate analysis [14]. Moreover, the common embedded methods for bioinformatic tasks are such as random forest, weight vector of SVM, and decision tree [4, 7], but they were not used in our work because filter and wrapper methods have been reported to be more stable for feature selection task [7].

In one of our previous studies [15], we combined IG (filter method) with a modified GA (wrapper method) to perform swine SNP selection. The IG ranked the SNPs for primary selection by an elbow method. The resulting group of the primarily selected features was then processed through two more selection steps by the modified GA and a frequency feature selection (FFS) method. We called the entire procedure as IG+modified GA+FFS. It was completely successful as it yielded a very small number of most statistically significant porcine-classifying SNPs which gave as highly accurate classification results as using all of the SNPs in the data set. Since our ultimate goal was to find the genes that are responsible for the differences between swine breeds, it was necessary to use SNPs data from as many breeds as possible. Therefore, we attempted to use the successful IG+modified GA+FFS on a more inclusive swine SNP data set in this study. This SNP data set included those of all swine breeds raised in countries in America, Europe, and Asia. The situation of a large number of SNPs and a small number of samples in this study was as challenging as that in the previous study, and the need to find a very small number of best PCSNPs that would provide the most accurate classification results were still the same. The aim of this study was to find a small number of PCSNPs that can accurately identify swine breed.

## Results

We propose a method that reduces the large number of porcine SNPs to a small number of statistically significant PCSNPs that can be used to successfully classify swine breed. Our study included investigation of several feature selection methods: IG, IG+GA, IG+modified GA, IG+GA+FFS, IG+FFS, and IG+modified GA+FFS as well as an SVM classification method. In this section, we present the results of SNP selection, principal component analysis (PCA), and identification of genes related to the selected SNPs.

### Experimental framework of feature selection and classification

The experimental framework of feature selection and classification consists of the following: 1) pre-processing and partitioning procedures, 2) perform feature selection procedures on the data sets, 3) perform classification procedures on the selected features. The end result of the experiment was a small number of the most significant PCSNPs that could identify a particular class of data (porcine breed) accurately. Incidentally, we used SVM as a classifier, which for the case of a lot of samples, the kernel would also take a lot of time to process [16]. For example, for Linear kernel, its computational complexity was $\mathcal {O}(m^{2}n)$, where *m* denotes the number of samples and *n* denotes the number of features. Hence, if the number of samples and features for training our model are reduced, the training time will be reduced as well.

The porcine SNP data needed to be pre-processed because there were some missing base-pairs in this real-world data set. Valid base-pairs are represented by 0, 1, or 2 in the data set, while the missing pairs are represented by −1. These missing pairs could confound the feature selection and classification procedures, leading to inaccurate classification. The pre-processing procedure was a single imputation method that estimated the missing values with a mode value. Then, the pre-processed data set was partitioned into training and test data sets. The training data set would be used for selecting features and training the classification model; the test data set would be used for testing the validity of the model. At the start of the feature selection procedure, the training data set was further partitioned into *r* randomly-seeded training sub-data sets and test sub-data sets. This round of partitioning was necessary because FFS needed to process a large number (*r*=10) of feature subsets in order to be able to select the most frequently occurred features that would be the most significant. In the feature selection procedure, IG ranked the features in each training sub-data set according to their classification significance and selects the upper-ranked features at and above a cut-point determined by an elbow method [15]. An elbow method is a method for interpretation and validation of consistency of clusters in a cluster analysis. It is used with a squared-error parameter to find the optimal number of clusters. Typically, this cut-point has to be set manually for IG to select a number of significant features which may not be automatically optimal, hence many cut-point values have to be tried and the resulting classification prediction needs to be observed which wastes a lot of time and resources [17]. It is most desirable to obtain the best cut-point automatically. A study has attempted to use *z*-score as an automatic method to find the optimal cut-point [18]. However, we used the elbow method in this study because it was able to give a low but sufficiently effective cut-point in our previous study [15]. The intermediate result from IG was *r*-ranked feature subsets which were passed along to FFS which would choose only high frequency features that appeared in every randomly-seeded training sub-data set on the basis that the higher the frequency, the more significant the feature would be.

For the IG+FFS method, the next step is then to further reduce the number of selected features by FFS. For the IG+modified GA+FFS, the number of dimensions of individuals in the modified GA is automatically set to be the same as the cut-point, and the next step is that the modified GA further selects the features provided by IG and sends a subset of them to SVM classifier. SVM evaluates the subset of features and then sends the evaluation result back to the modified GA. This step computes iteratively until the specified maximum number of generations of GA individuals is reached, resulting in the best subsets of features in terms of classification result determined by linear and radial basis function (RBF) kernels of SVM. These two best subsets are then sent to FFS.

**Fig. 1:**
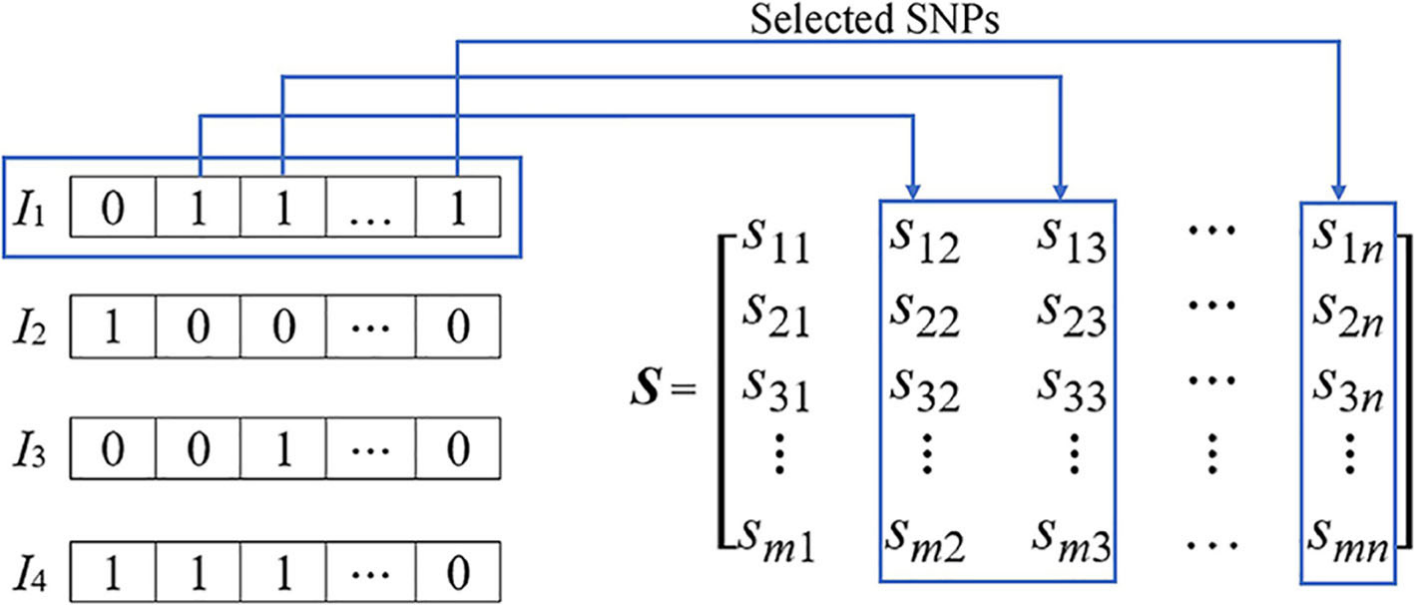
Examples of four modified GA individuals and their connection with matrix *S*

**Fig. 2:**
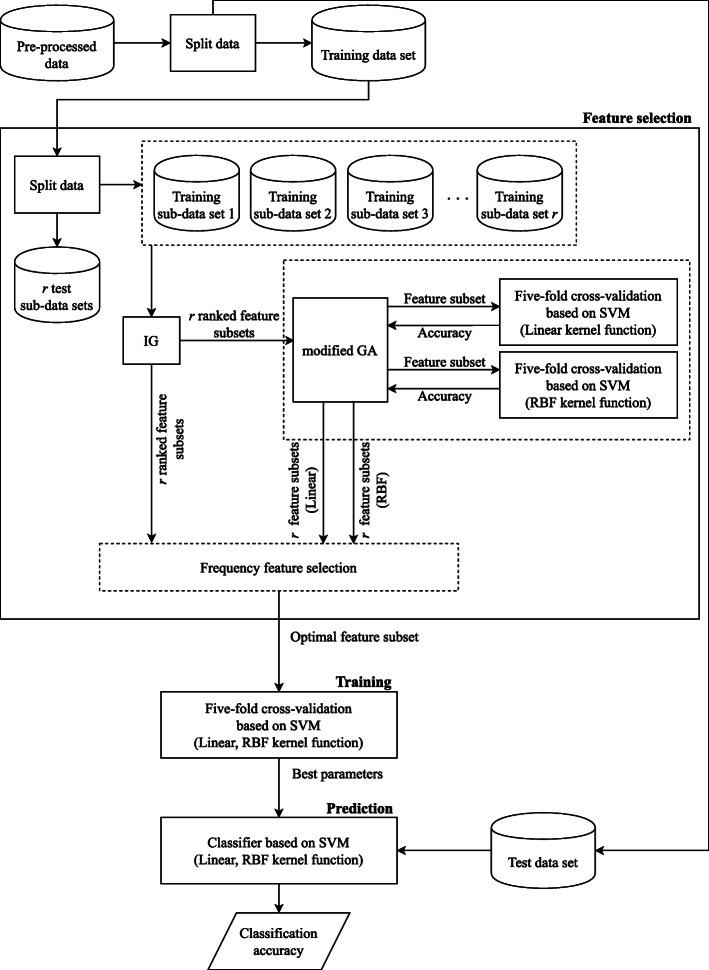
The framework of experiments on the IG+FFS and IG+modified GA+FFS in combination with SVM methods

In this study, the SNP data (*S*) were represented by an *m*×*n* matrix, where *n* is the number of dimensions of each individual and *m* is the number of swine samples. Figure 1 illustrates examples of *n* SNP column vectors from a matrix of *m* swine samples (*S*_*mn*_) and four modified GA individuals derived from them. When the slot at a position of a certain dimension has a value of 1, the column vector, representing an SNP, in the matrix *S* corresponding to that position is selected. For example, for *I*_1_, the selected column vectors are vectors in columns 2, 3, and *n* which will be iteratively evaluated of their classification accuracy by linear and RBF kernels of SVM until the maximum number of generations in modified GA is reached. Next, two subsets of selected features from linear and RBF kernels are processed by FFS that combines them and further selects only a small number of high frequency features into an optimal subset. Of note here is that, in the actual experiment, we compared this subset of selected features with the subset obtained from IG alone and found that the subset obtained from IG alone was much bigger, indicating that FFS was truly effective in selecting only a few high frequency features. This is the end of the feature selection step illustrated in Fig. 2. Next, the model is further trained with the columns (in the entire training data set) that correspond to the optimally selected features from the feature selection step. Again, five-fold cross-validation was employed to obtain a set of optimal model parameters. Then, the optimal model was tested with the test data set in the prediction step to find its classification accuracy. The experiment was run 10 times with 10 different randomly-seeded data sets.

### Experimental setup

The swine sample data set was randomly-seeded into 10 training data sets and 10 test data sets to increase the reliability of the experiment. The ratio of the number of swine samples in all of the training data sets to that in all of the test data sets was 80:20.

The initial values of the parameters for IG+GA, IG+modified GA, and IG+modified GA+FFS methods were set as follows: a population size of 30 chromosomes. The reason that we set the population size to be 30 was that a higher number would result in too large a number of features that would incur a lot of wasted computational time. We did not choose to investigate other smaller population sizes because several studies, briefly described below, have investigated them already and suggested that a population size of at least 20 was necessary and a population size of 30 was used in at least two studies. Roeva et al. investigated the cases of 5, 10, 20, and 30 chromosomes and GA, and reported that at least 20 chromosomes were necessary for achieving a better solution [19]. For Particle Swarm Optimization, Chen et al. reported that a larger population size of *n*≥30 made it converged faster [20]. Lastly, Rodrigues et al. have done a SNP selection study similar to ours and reported that he also used a population of 30 [10].

**Fig. 3:**
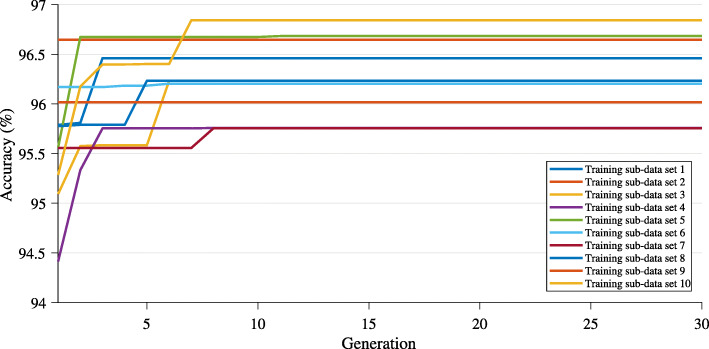
Classification accuracy versus number of generations of population in modified GA

The crossover rate (*P*_*c*_) of 0.8; mutation rate (*P*_*m*_) in the range of 0.1–0.9; the maximum number of generations of 10. The number of generations was set as 10 because preliminary trial runs showed that GA met its stop criterion within 10 generations and setting it to a higher number was not likely to increase the accuracy at all. Our method converged before 10 generations in 30 runs. We show a graph that extended to 30 generations in Fig. 3 in this paper so that readers can see that nothing changed beyond 10 generations. The initial values of the parameters of SVM were set as follows: a *C* in the range of 10^−6^−10^6^ and a *γ* of RBF kernel in the range of 10^−10^−10^10^. The selection threshold of the FFS method was set at 9 from trial and error.

**Fig. 4:**
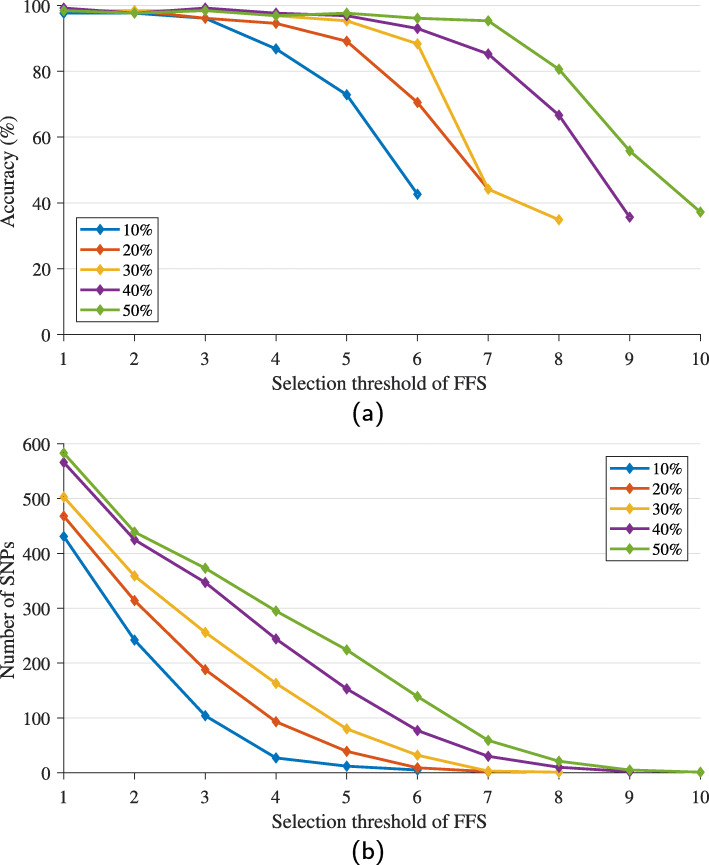
Effects of the assigned percentage of selected features by GA in the initialization step on the final number of selected PCSNPs by IG+GA+FFS method (**b**) and their classification accuracy (**a**) that vary with FFS selection threshold

It is noted that the chance to obtain PCSNPs is proportion to the percentage of randomly generated 1 values for selecting SNPs. In the proposed method, we did not control this, but it makes use of IG ranking process of SNPs by IG in combination with the process of screening out some SNPs by the elbow method. Therefore, it can automatically select the initial SNPs instead of hard threshold. However, we have tried to use the original GA in the IG+GA+FFS hybrid to reduce the number of features in the population initializing step to 10–50% of the total number of features. The results of the runs are shown in Fig. 4. SNPs were able to get selected with the frequency of 6–10 at most (from runs of 10 randomly seeded training sub-data sets) for the population initializing of 10–50%, respectively, as shown in Fig. 4a. This can be attributed to the feature selection procedure of GA. If the procedure randomly generated few 1 values for selecting SNPs, i.e. few SNPs were selected initially, the chance for the method to find and select a high number of statistically significant PCSNPs was low as shown in Fig. 4b.

### Results of SNP selection and swine breed classification

For comparison between all five feature selection method—IG+GA, IG+modified GA, IG, IG+FFS and IG+modified GA+FFS, we used the optimum parameter values in each method. In 10 runs, several values of various parameters were involved. Here, we reported the best parameters from the majority of runs out of 10 runs. The best *C* parameter for linear kernel after the training was 10^−1^. The best *C* and *γ* parameters for RBF kernel were 10^6^ and 10^−7^. The result—the mean number of selected SNPs obtained from every method and the mean classification accuracy obtained by using the features selected by each method—are shown in Table 1. Every method selected nearly the same number of SNPs: 2.03%, 3.04%, 4.05%, 2.36% and 1.62% of the whole SNPs in the data set, respectively, where IG+modified GA+FFS selected the least number of PCSNPs. Nevertheless, it did not give the highest mean accuracy values (94.81% for the linear kernel and 95.12% for RBF kernel that IG+FFS achieved (95.66%). The results achieved by IG+GA+FFS are not shown because the approach was not able to select more than a few SNPs since the frequencies of occurrences of most SNPs were below the specified threshold. The accuracy values achieved by all of the methods were tested by a one-way ANOVA analysis whether the differences between them were statistically significant. Generally, one-way ANOVA is used for comparing more than two means whether at least a pair of the means are significantly different or not. In our case, the ANOVA results indicated that the differences were not significant at *p*>0.05 (Table 2 where *p*=0.73). If the *p*-value from a statistical analysis is less than or equal to the set significance level, the data is considered statistically significant. The widely-accepted significance level (or alpha) is 0.05, Hence, it was concluded that IG+modified GA+FFS was the best feature selection method among these five methods because it provided the smallest number of PCSNPs and gave a good accuracy value that was not statistically different than the best accuracy value achieved by any of the five methods. On top of that, this accuracy value was also not statistically different from the accuracy value obtained from using the whole SNPs.

**Table 1:** The mean number of finally selected PCSNPs by each of the five feature selection methods and their resulting accuracy as well as the accuracy provided by using the entire swine SNPs in the data set

Method	PCSNPs		Accuracy (%)	
	Linear	RBF	Linear	RBF
Whole SNPs	10,210	10,210	**95.66*±*1.28**	**95.66*±*1.28**
	(100%)	(100%)		
IG+GA	207.70 ±42.71	209.90 ±37.57	95.27 ±1.57	94.88 ±1.11
	(2.03%)	(2.06%)		
IG+modified GA	319.10 ±104.02	310.70 ±84.89	95.74 ±1.47	95.35 ±1.42
	(3.13%)	(3.04%)		
IG	413.30 ±88.22	410.30 ±88.22	95.43 ±1.53	95.58 ±1.51
	(4.05%)	(4.05%)		
IG+FFS	240.80 ±15.33	240.80 ±15.33	95.58 ±1.27	**95.66 ±1.22**
	(2.36%)	(2.36%)		
IG+modified GA+FFS	**164.90*±*36.11**	**164.90*±*36.11**	94.81 ±1.46	95.12 ±1.55
	**(1.62%)**	**(1.62%)**

**Table 2:** One-way ANOVA results of the significance difference between mean accuracy values obtained from using the whole features in the data set and from using only the features selected by various selection methods

Source	Sum of squares		Degrees of freedom		Mean square		*F*-statistic		*p*-value	
	Linear	RBF	Linear	RBF	Linear	RBF	Linear	RBF	Linear	RBF
Methods	5.82	5.13	5	5	1.16	1.03	0.57	0.56	0.73	0.73
Error	111.11	99.39	54	54	2.06	1.84	-	-	-	-
Total	116.93	104.52	59	59	-	-	-	-	-	-

Regarding the resulting number of selected SNPs, IG+GA would reduce the number of SNPs to a half regardless of the value of *P*_*m*_. In contrast, the number of PCSNPs selected by IG+modified GA was highly sensitive to the value of *P*_*m*_.

**Fig. 5:**
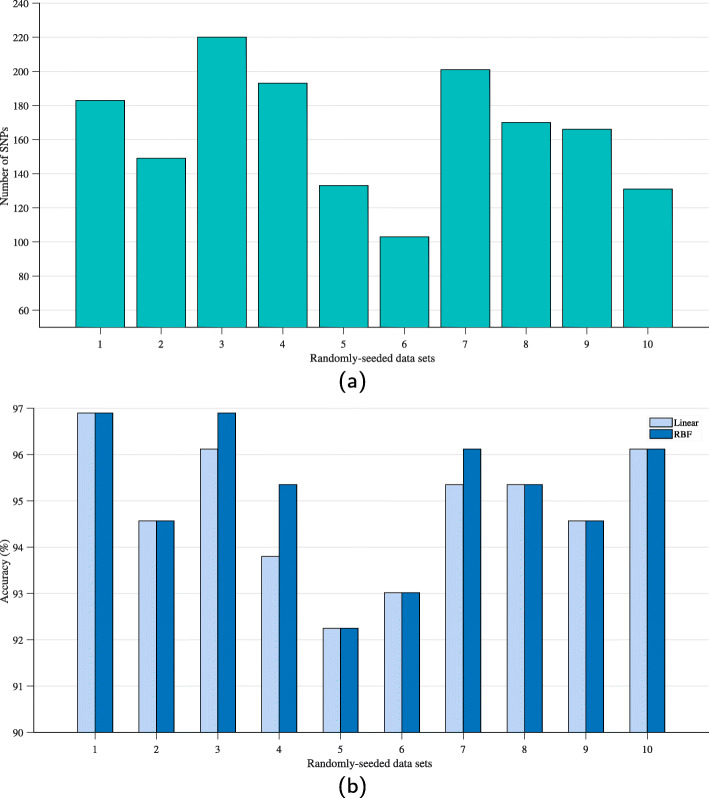
**a** The numbers of selected PCSNPs obtained from 10 randomly-seeded data sets; **b** the classification accuracy values obtained from using the selected PCSNPs from those data sets

The numbers of selected PCSNPs from 10 randomly-seeded data sets by IG+modified GA+FFS are shown in Fig. 5a, and the resulting classification accuracy provided by them are shown in Fig. 5b. The 1st set of 183 PCSNPs achieved the highest classification accuracy (96.90%), so it was brought to use in the principal component analysis (PCA) of swine breeds to see whether this set of PCSNPs can truly and clearly classify a group of swine samples into different breeds. The classification experiment in this study was done on 10 randomly-seeded data sets because we wanted the results to be most reliable. Our decision was justified as can be seen in the 5% difference in the classification accuracies obtained from the first and the fifth training and test sets by the IG+modified GA+FFS, shown in Fig. 5. The much lower accuracy obtained from the fifth training and test data sets might be because of the training data set not including a high enough number of PCSNPs.

**Fig. 6:**
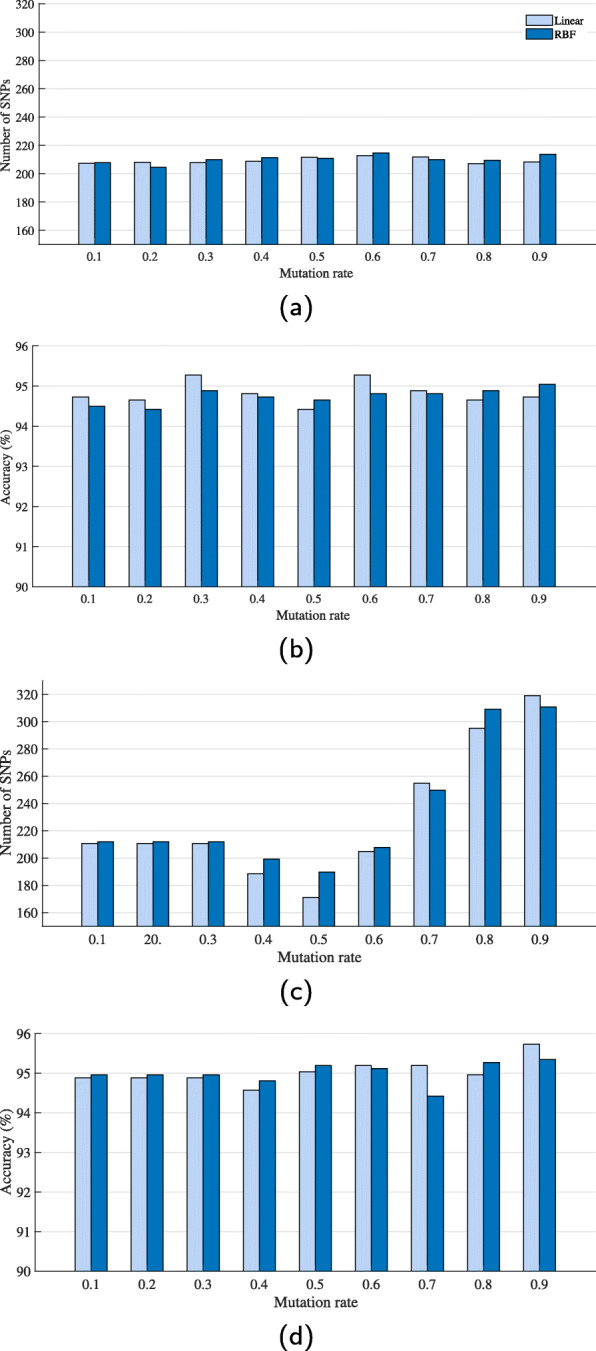
Fig. 6 Final mean number of selected PCSNPs (**a** and **c**) and the classification accuracy (**b** and **d**) obtained from the selected PCSNPs from 10 runs of different randomly-seeded data sets; (**a**) and (**b**) are from IG+GA while (**c**) and (**d**) are from IG+modified GA

The comparative numbers of selected PCSNPs achieved by IG+GA and IG+modified GA and the mean classification accuracy achieved by these selected features as *P*_*m*_ was varied from 0.1-0.9 are shown in Fig. 6. It can be seen in Fig. 6a that the numbers of selected PCSNPs by IG+GA as *P*_*m*_ was varied from 0.1 to 0.9 were not very different at all, and these numbers were about one half of the number of PCSNPs first selected by IG which is in a good agreement with the results from [15]. In addition, it can be seen in Fig. 6b that the value of *P*_*m*_ that provided the best accuracy (95.27%) from the linear kernel was 0.3, while the *P*_*m*_ value that provided the best accuracy (95.04%) from the RBF kernel was 0.9. As for the numbers of selected PCSNPs by IG+modified GA as *P*_*m*_ was varied, the value of *P*_*m*_ that gave the best accuracy results was 0.9 which gave 95.74% and 95.35% accuracy from the linear kernel and RBF kernel, respectively, as can be seen in Fig. 6d, while the mean numbers of selected PCSNPs were 319.10 and 310.70, respectively, as shown in Fig. 6c. Therefore, for the subsequent experiment, a *P*_*m*_ value of 0.9 was also used for IG+modified GA+FFS.

### Results of the PCA analysis

**Fig. 7:**
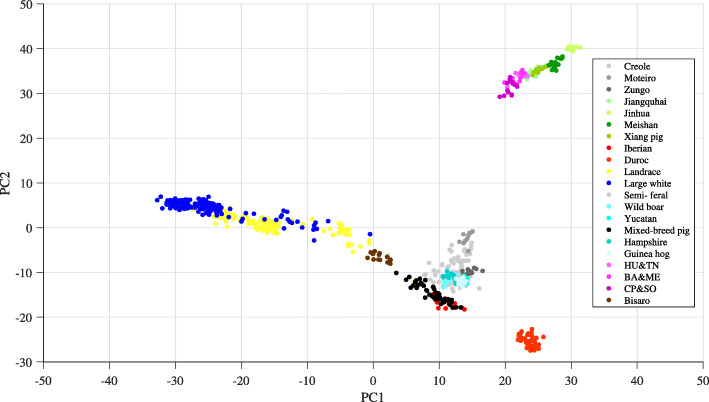
Conventional PCA projection from using the entire SNPs in the data set

The PCA result of the entire collection of SNPs in the data set is shown in Fig. 7. The figure shows the relationship between the principal components PC_1_ and PC_2_ which are the top two PC _*s*_. It can be seen that the analysis was able to distinguish the following swine breeds: Chinese pig, Vietnam pig, Landrace, Large white, Mixed-breed pig, Iberian, Bisaro, and Duroc. This result agrees very well with the PCA results in [15] and [21], i.e., all of those classification results of the following swine breeds were in complete agreement: Chinese pig (green), Iberian (red), Bisaro (brown), Landrace (yellow), Large white (blue), and Duroc (orange). The additional Asian swine breeds that were included in the new data set that we used were also clearly classified: Vietnamese pig (pink) [22] and Mixed-breed pig (black). However, the swine breeds in the group of village pigs [21] have been crossed extensively with each other and so their classification results were overlapped to some extent (Fig. 7).

**Fig. 8:**
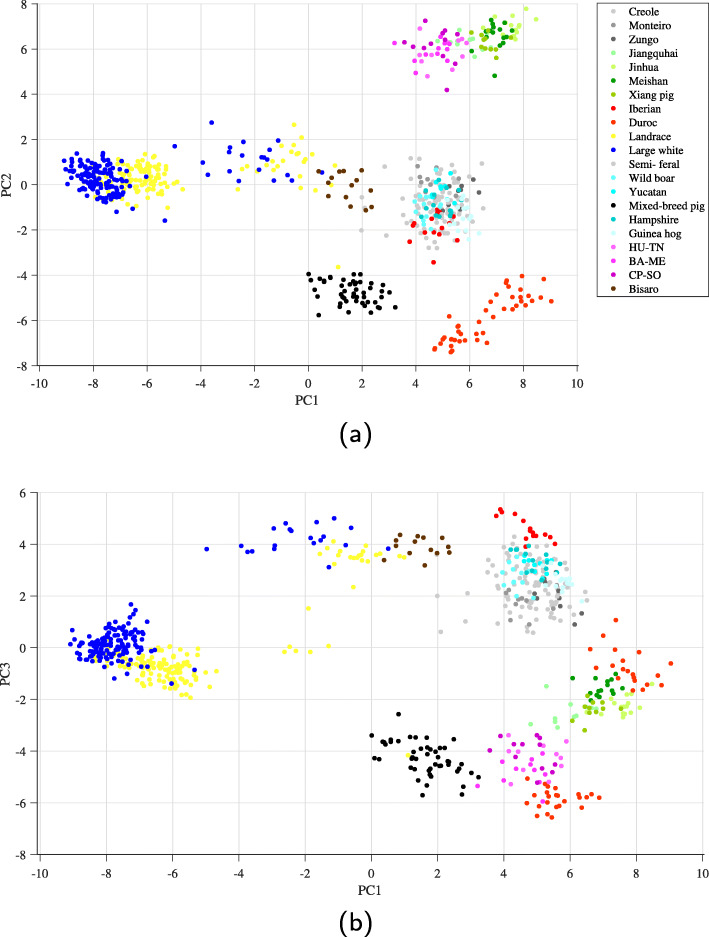
Conventional PCA projection from using only 183 PCSNPs in the data set. **a** PC1-vs-PC2 and **b** PC1-vs-PC3

It can be seen in Fig. 8a (the relationship between the principal components PC_1_ and PC_2_) and Fig. 8b (the relationship between the principal components PC_1_ and PC_3_ where PC_3_ is the third PC from the top that has the highest variance) that the results of an SNP analysis by PCA by using only 183 PCSNPs (obtained from one of the randomly-seeded data sets that provided the highest accuracy) that were selected by our proposed method were virtually the same as those from the analysis that used the whole 10,210 SNPs in the data set of which details are shown in Table 6.

### Results of identification of genes related to the selected sNPs

The gene identification was done by inputting the union of 341 selected PCSNPs from 10 randomly-seeded data sets (See Supplementary Table S1, Additional file 1) into an application called genome build 2.0 that searched for the genes related to them. The search found 248 genes, and their gene expression pathways were further searched for in a Protein ANalysis through an Evolutionary Relationships (PANTHER) biological database of gene families that can classify gene products and indicate their function. We found pathways of 94 genes as shown in Table 3 that includes Gene symbol, Chromosome (Chr), and MAPINFO. The gene symbols 1–6, 7–8, 9–19, 20–33, 34–45, 46–51, 52–59, 60–65, 66–70, and 71–94 represent the genes containing the PCSNPs that were found to occur at the highest (10) to lowest (1) frequency in that order in the 10 randomly-seeded data sets. In addition, Table 4 shows the function of the gene products of the first five genes listed in Table 3. For the gene in the 6^th^ order (RTN3), although it was found in the 10 randomly-seeded data sets, but its gene function was not found in PANTHER pathway. However, we have found a report about RTN3 in [23] that RTN3 is one of 26 genes in a functional network that can indicate meat quality. Top functions of this gene network are lipid metabolism, small molecule biochemistry, and molecular transport.

**Table 3:** Discovered gene families from the final selected PCSNPs

No.	Gene symbol	Chr	MAPINFO	No.	Gene symbol	Chr	MAPINFO	No.	Gene symbol	Chr	MAPINFO
1	PTPRK	1	38598147	33	GALNT12	1	268890145	65	HES1	13	140688388
2	ABCA5	12	11388469	34	FIGN	15	75340693	66	CCDC13	13	28893435
3	SEMA3E	9	106779607	35	BMPR1B	8	133950496	67	KIT	8	43651639
4	KCNU1	15	14889528	36	PPEF2	8	75662581	68	GRK5	14	140846738
5	SLC28A3	10	34818681	37	GNAT3	9	110416160	69	PIK3C3	6	118157644
6	RTN3	2	7728326	38	CD3E	9	50672784	70	PDE4B	6	134910792
7	SORCS3	14	125978735	39	PSAP	14	80626307	71	LOC100153360	1	313111618
8	DBH	1	307192626	40	SNCA	8	138635995	72	LOC100739240	3	74337201
9	SNTB1	4	19453169	41	HACE1	1	80302938	73	NCR2	7	41832022
10	VAT1L	6	10041550	42	TRHR	4	30842196	74	CDK8	11	3651117
11	LOC100622482	6	82916168	43	NTS	5	101073253	75	SATB1	13	6000732
12	CUEDC1	12	35017301	44	ADRA1B	16	69129145	76	ROR2	14	3557219
13	CALB2	6	13899346	45	RXRG	4	93070713	77	TRPM2	2	143991472
14	MACROD1	2	7103886	46	AAAS	5	18962460	78	CAPZB	6	71859152
15	KLHL25	7	93415034	47	NEK2	9	144617825	79	ANKRD35	4	109093503
16	GRK5	14	140846738	48	RNF180	16	45700636	80	SECISBP2L	1	136455430
17	DPEP1	6	504970	49	EML5	7	117152298	81	LMX1B	1	301126002
18	LOC100155953	7	122798672	50	ABLIM1	14	135899761	82	DTL	9	144214338
19	ZMIZ1	14	88275273	51	RBM19	14	40500953	83	PPP2R5A	9	144185861
20	LOC100156904	1	296533542	52	PRUNE2	1	256372239	84	RCAN1	13	208012602
21	PCDH15	14	104808991	53	PDZK1IP1	6	119087839	85	RAPGEF4	15	24972365
22	SLC22A5	2	140066357	54	GAD2	10	54668661	86	LHX2	1	298735016
23	LNX1	8	42621415	55	CP	13	97407074	87	IQSEC3	5	69759629
24	DNAJB12	14	81222592	56	SAMD3	1	36604527	88	LY96	4	67548067
25	CDKAL1	7	17100569	57	SLC35F4	1	207232466	89	WHAMM	7	57639263
26	CRB2	1	297932234	58	FCRLB	4	96854257	90	CHD1L	4	110076256
27	SPOCK2	14	80904334	59	ENPP5	7	47241389	91	ADAMTS16	16	82812184
28	CCND2	5	68326348	60	CYP7B1	4	75934281	92	TBC1D14	8	3354915
29	TXNDC15	2	142718262	61	AGRP	6	25411042	93	PARM1	8	74934147
30	FRAS1	8	77822157	62	NOX4	9	25460973	94	FGFR1	15	55262655
31	A2M	5	65318067	63	LOC100511652	9	12772773				
32	STAT3	12	20767800	64	ARHGAP26	2	150907623

**Table 4:** Functions of gene products

No.	Gene symbol	Gene ontology biological process complete
1	PTPRK	transforming growth factor beta receptor signaling pathway (GO:0007179);
		negative regulation of keratinocyte proliferation (GO:0010839); cell migration (GO:0016477);
		negative regulation of cell migration (GO:0030336); protein localization to cell surface (GO:0034394);
		cellular response to reactive oxygen species (GO:0034614); cellular response to UV (GO:0034644);
		peptidyl-tyrosine dephosphorylation (GO:0035335); negative regulation of cell cycle (GO:0045786);
		negative regulation of transcription; DNA-templated (GO:0045892); focal adhesion assembly (GO:0048041)
2	ABCA5	negative regulation of macrophage derived foam cell differentiation (GO:0010745);
		cholesterol transport (GO:0030301); cholesterol efflux (GO:0033344);
		high-density lipoprotein particle remodeling (GO:0034375); transmembrane transport (GO:0055085)
3	SEMA3E	branching involved in blood vessel morphogenesis (GO:0001569);
		negative regulation of cell-matrix adhesion (GO:0001953);
		sprouting angiogenesis (GO:0002040); regulation of cell shape (GO:0008360);
		negative regulation of angiogenesis (GO:0016525); synapse organization (GO:0050808);
		negative chemotaxis (GO:0050919); semaphorin-plexin signaling pathway (GO:0071526);
		regulation of actin cytoskeleton reorganization (GO:2000249)
4	KCNU1	potassium ion transport (GO:0006813); ion transmembrane transport (GO:0034220);
		potassium ion transmembrane transport (GO:0071805)
5	SLC28A3	pyrimidine nucleobase transport (GO:0015855); purine nucleoside transmembrane transport
		(GO:0015860);
		pyrimidine nucleoside transport (GO:0015864); sodiumion transmembrane transport (GO:0035725);
		pyrimidine-containing compound transmembrane transport (GO:0072531); purine nucleobase
		transmembrane transport (GO:1904823)

A piece of information that supports our valid PCSNPs results is that the PTPRK gene that is related to the most frequently selected and highest-ranked SNP from all 10 randomly-seeded data sets that we found has been reported to be differentially expressed in two swine groups: a group of adult and juvenile swine with Rapacz familial hypercholesterolemic and a group of WT swine, as indicated by the results of a microarray analysis [24]. Lee et al. analyzed the gene ontology of Landrace pigs and reported that PTPRK gene contained PCSNPs in the case of under-dominance in the final weight and over-dominance in the backfat thickness [25]. Lastly, LOC100511786, LOC100625374, and LOC100515404 are examples of genes containing three selected PCSNPs of which frequencies of occurrences were 10–found in all 10 randomly-seeded data sets apart from the first six genes listed in Table 3. Surprisingly, these genes were not found in a PANTHER search. All genes of which frequencies of occurrences were 8 and up to 10 are shown in Table 5. It is quite possible that they may be important genes of which functions have not been hitherto discovered–investigation into them may provide lucrative information.

**Table 5:** Discovered genes that did not match any genes in the PANTHER database

No.	Gene symbol	No.	Gene symbol	No.	Gene symbol
1	LOC100511786	14	DLK1	27	LOC100516653
2	LOC100625374	15	LOC100738463	28	LOC100628179
3	LOC100515404	16	LOC100157816	29	AGMO
4	PTPN3	17	ITGB5	30	LOC100127144
5	LOC100737182	18	LOC100512373	31	LOC100154421
6	LOC100153068	19	LOC100511786	32	LOC100525245
7	DLK1	20	LOC100513826	33	LOC100624347
8	TLL1	21	LOC100625374	34	LOC100515332
9	LOC100738463	22	LOC100628176	35	LOC100622308
10	ITGB5	23	LOC100622588	36	BRP44L
11	LOC100156777	24	LOC100627046	37	TCF4
12	LOC100512373	25	C13H21orf63	38	LOC100736576
13	CTNNA2	26	LOC100519752

**Table 6:** Details of swine samples in the data set used in this study

Breed	Location	Number of samples
Creole	Alto Baudo-Colombia, Baja Verapaz-Guatemala, Granma-Cuba, Guanacaste, Alajuela-Costa Rica,	
	Loja-Ecuador, Misiones-Argentina, Pinar del Rio-Cuba, Titicaca area-Peru	90
Monterio	Pocone-Brazil	10
Zungo	Cerete-Colombia	10
Jiangquhai	China	11
Jinhua	China	16
Meishan	China	16
Xiang pig	China	11
Iberian	Spain	15
Duroc	Denmark, Holland, USA, Thailand*	44
Landrace	Denmark, Holland, USA, Thailand*, Hanoi-Vietnam**	146
Large white	Denmark, Holland, USA, Thailand*	149
Semi- feral	Formosa-Argentina	10
Wild boar	Hungary, Poland, Tunisia	13
Yucatan	Indiana-USA	10
Hampshire	UK, USA	14
Guinea hog	USA	15
Bisaro	Portugal	14
Mixed-breed pig	Thailand*	48
HU-TN	Vietnam**	11
BA-ME	Vietnam**	11
CP-SO	Vietnam**	12

Note: * indicates that the swine samples are from Thailand Pig data set [28]; ** indicates that the samples are from [22]; the rest of the samples are from [21]

## Discussion

In this section, we first discuss the parameter tuning results for IG+GA and IG+modified GA and the final number of selected features as well as their classification accuracy. Then, we discuss SNP selection by IG alone and by IG+modified GA+FFS. Lastly, we discuss breed identification by our PCA analysis.

### SNP selection and swine breed classification

Firstly, we ran IG+GA and IG+modified GA with varying values of *P*_*m*_ to find the optimum value of *P*_*m*_ for our final experiment and found that, for IG+GA, no matter what value of *P*_*m*_ was set and which of the 10 randomly-seeded data sets the method acted on, the number of features selected by this method was almost the same, around a half of the number of all features, whereas the IG+modified GA was sensitive to *P*_*m*_. This tuning result was reasonable because the mutation operator of a conventional GA flips a ‘0’ or ‘1’ bit with an equal probability; hence, the mutation does not affect the number of selected features in any ways. However, the mutation probability for flipping 0 bit to 1 bit of modified GA is not equal to the probability of flipping 1 bit to 0 bit. These probabilities vary with the value of *P*_*m*_, and so a smaller or larger number of selected features can be set via a particular value of *P*_*m*_. Nevertheless, an optimum value of *P*_*m*_ also depends on the classification accuracy obtained from the set of selected features, so we can have a degree of control over the number of selected features by varying the *P*_*m*_, but we cannot vary it to an arbitrary value as we are pleased. The true optimum *P*_*m*_, 0.9, was found only by also performing classification, i.e., the whole procedure. Therefore, for the subsequent experiment, a *P*_*m*_ value of 0.9 was also used for IG+modified GA+FFS. It should be noted, as can be seen in Fig. 6c and d, that for the values of *P*_*m*_ of 0.1–0.3, the mean numbers of selected PCSNPs and the accuracy values that they produced were the same because the initial population already provided the best results and the low mutation rate did not alter the outcomes in any which ways, i.e, the numbers of selected PCSNPs were so low that the classification accuracy values provided by the later mutated generations could not improve them any further.

**Fig. 9:**
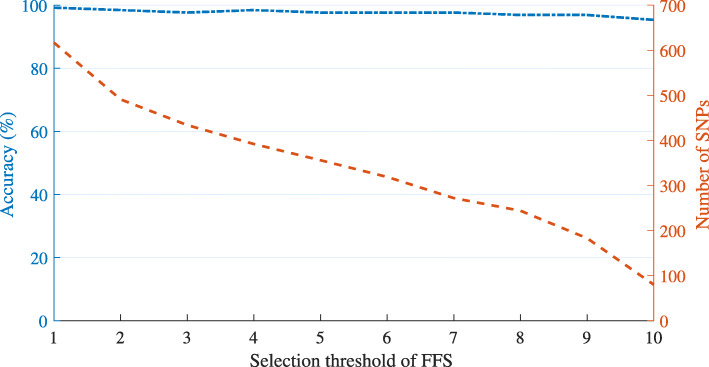
Effects of FFS threshold on classification accuracy and the final number of PCSNPs selected by IG+modified GA+FFS

**Fig. 10:**
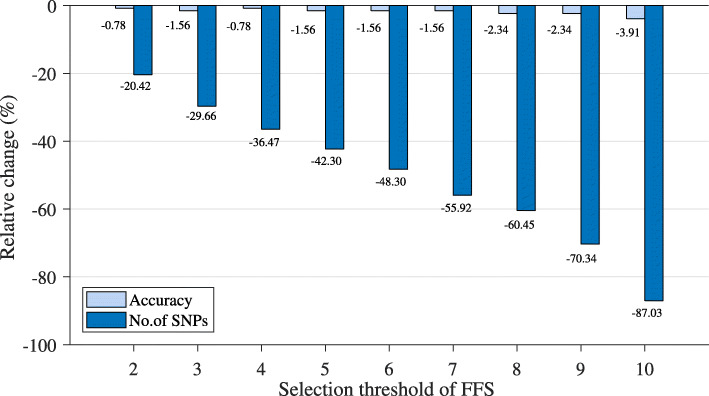
Relative differences of classification accuracy and number of selected SNPs by IG+modified GA+FFS with different values of FFS threshold with respect to FFS threshold equal to 1

In addition, in the final runs, we assigned the threshold value for FFS as 9. We had investigated lower and higher values of this threshold, from 1 to 10, and selected 9 as an optimal value. The rationale behind our selection is explained in the following passage. Figure 9 shows the selected PCSNPs from setting the FFS threshold from 1 to 10 on the first randomly seeded data set. It can be seen that the lower the threshold, the higher the number of selected PCSNPs, while the classification accuracy stayed the same or changed slightly as the threshold varied. Figure 10 shows that the accuracy values from setting the threshold from 1 to 10 differed by only 3.91%, but the number of selected PCSNPs differed by as much as 87.03%, demonstrating that setting a high threshold value that results in a smaller number of most significant PCSNPs is still able to achieve high classification accuracy. Setting the threshold to 10 provided a smaller number of selected PCSNPs than setting it to 9 (that provided a -2.34% relative difference in accuracy compared to the threshold value of 1 that provided the best accuracy but did not provide a small enough number of selected PCSNPs), but the classification accuracy that it provided was lower (-3.91% relative difference in accuracy). Similarly, setting the FFS threshold value to 9 rather than 8 provided an identical classification accuracy but a lower number of selected PCSNPs.

As shown in Table 1, the classification accuracy values achieved by every method were not significantly different at *p*>0.05, but the number of selected features were different. The difference stemmed from the original feature selection method rather than the combined FFS; that is, IG alone selected a mean number of 413.30 features while IG+modified GA selected 310.70 features, and after these features were further selected by FFS, the difference still remained; IG+FFS selected 240.80 features while IG+modified GA+FFS selected 164.90 features.

### Information gain values and PCSNPs selected by IG and IG+modified GA+FFS methods

**Fig. 11:**
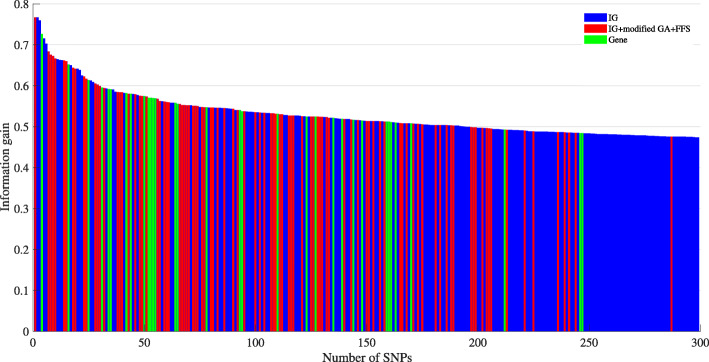
Plot of information gain value versus number of selected PCSNPs by IG (blue bars), IG+modified GA+FFS (red bars) and the genes found in the PANTHER pathway (green bars)

A plot of information gain values versus the selected PCSNPs by IG and IG+modified GA+FFS methods on a selected randomly-seeded data set is shown in Fig. 11. The SNPs that were selected by IG alone are shown as blue bars while those selected by IG+modified GA+FFS are shown as red bars. In addition, the green bars in Fig. 11 represent the selected 33 PCSNPs of which genes containing them were found in PANTHER pathway. First of all, it can be seen that IG+modified GA+FFS selected fewer PCSNPs than IG alone did as we had expected. Secondly, IG+modified GA+FFS selected not only the PCSNPs that had a high information gain value but also a few of those with a relatively low information gain value, signifying that the PCSNPs could be lower-ranked PCSNPs with respect to information gain value, indicating that IG alone was not able to select some PCSNPs that IG+modified GA+FFS was able to. This is supported by the fact that there exist real genes in the PANTHER pathway that were identified by these relatively lower-ranked PCSNPs. This kind of discovery of lower-ranked yet significant PCSNPs is supported by [26].

**Fig. 12:**
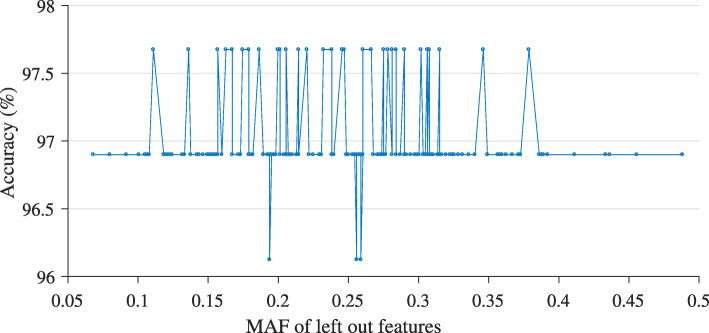
Performance of the model versus their MAF value when each PCSNP is left out

In addition, minor allele frequency (MAF) is the frequency of the second most frequent allele for a given SNP. A low MAF may imply that a major allele for the SNP is conserved and more or less fixed, but not necessarily. This measure indicates the degree of variation of genotypes for a given SNP in a given population. In other words, it gives an idea about how common the SNP is. MAF helps to differentiate the common and the rare SNPs in a population. Kasamo et al. found mutated genes with SNPs that had an MAF of lower than 1% that caused chronic progressive external ophthalmoplegia symptoms [27]. If a SNP has a lower-than-1% MAF, that SNP is a mutated SNP. We hypothesized that a SNP with a low MAF was likely to differentiate porcine breeds well. If we left it out from a run, the classification accuracy should decrease. Therefore, we did leave-one-SNP-out experiments and plotted the obtained accuracy values against the MAF value of each SNP. The graph in Fig. 12 shows that there were only 3 instances (3 PCSNPs) when the accuracy value decreased. Those 3 PCSNPs—ALGA0114715_T, ASGA0001200_A, and ALGA0001286_T—had an MAF in the range of 0.19–0.26 (higher than 1%). Therefore, those PCSNPs were not mutated SNPs, but they were certainly PCSNPs. Hence, all PCSNPs should be taken into account to classify these porcine breeds.

### PCA analysis

According to PCA, the swine breed classification results from SNPs indicate that Landrace, Large white, and Duroc breeds that have been farmed globally and widely used as parent breeding stock still retain their pure breed even though they have been farmed in numerous regions in the world. Iberian and Bissaro which are outgroup pigs were clearly separated from village pigs [21]. Chinese pigs that are included in this data set–Jiangquhai, Jinhua, Xiang pig, and Meisha–showed their Chinese traits clearly. A PCA analysis by Ishihara et al. [22] showed that 90 samples of Vietnamese pigs (Figure 1 in [22]) of 15 native breeds which were clearly different genetically and 6 samples of Landrace breed fell into 3 main groups: groups from the northern region, central region, and southern region, indicating that the breeds of Vietnamese pigs may be closely related to the geographical locations where they were farmed. Some Vietnamese breeds overlapped with Landrace breed which might be the result of cross-breeding. Our analysis results (in Fig. 8a and b) show that HU-TN, BA-ME, and CP-SO Vietnamese breeds are closely related and form their own distinct group. Their locations in the PCA plot are close to the locations of Chinese pigs. For the new mixed-breed pigs in Thailand that are resistant to stress, provide a lot of red meat, and consistently produce high-quality offspring, their locations in our PCA plot were clearly separate from those of the other breeds (see Fig. 8a and b). All of these pieces of information indicate that the breeds of pigs of which data had been collected have been appropriately developed to retain their breed purity. Lastly, the small number of the best PCSNPs that can differentiate swine breeds found by our developed selection method should render an identification of the genes related to these PCSNPs more achievable and less expensive, i.e., more practical.

## Conclusions

To conclude, this work attempted to select and classify a small number of the best porcine-classifying SNPs (PCSNPs) for differentiating swine breeds. The feature selection methods used were IG, IG+GA, IG+FFS, IG+modified GA, and IG+modified GA+FFS and the classification method was SVM. The IG+modified GA+FFS was able to find the smallest number of the most PCSNPs with the highest classification accuracy. It was 1.62% of the whole SNPs in the data set that provided 95.12% classification accuracy. This method had already been used successfully in one of our previous studies on a smaller data set and continued to perform effectively on a bigger data set in this study. These selected PCSNPs were then put through a search in the PANTHER database to find genes related to them. As a result, 94 genes were found that will benefit future swine breed improvement.

## Methods

### The data set of sNPs used in this study

The details of the data set used in this study is shown in Table 6. It had 676 swine samples of 21 breeds with 10,210 SNPs. The swine samples in this data set were chosen and collected from 3 established data sets: a Porcine colonization of the Americas data set [21] which had 315 swine samples of 17 breeds such as Creole, Iberian, Jinhua, Duroc, Landrace, and Large White; a data set of pigs raised in Thailand [28], which had 321 swine samples of 4 breeds–Duroc, Landrace, Large white, and mixed-breed pig; and a data set of pigs raised in Vietnam [22] which had 40 swine samples of 4 breed–HU-TN, BA-ME, CP-SO, and Landrace. The collected data in our data set had been processed through a quality control procedure that utilized a PLINK computer program, but there were still some missing values. These values were then estimated by a mode value in a single imputation method. The data set is available for download at https://github.com/dsmlr/th-vn-us-swine.

Selection of a small number of the most significant features for classification is very important because even though gene and SNP matching procedure is adequately efficient, the validation procedure for each match is costly and so least significant SNPs are preferably not included. In [15], a combination of filter and wrapper methods plus a frequency feature selection (FFS) method were successfully used for single nucleotide polymorphisms (SNPs) selection. This information gain+modified genetic algorithm+frequency feature selection feature selection method was also used in this study but on a larger swine SNP data set. In the section below, this selection method is described in more detail. In addition, the support vector machine (SVM) that was used both in [15] and this study is also described and explained.

### Related works and basic concepts on information gain, genetic algorithm, and support vector machine

The information gain (IG)+modified genetic algorithm (GA)+frequency feature selection (FFS) hybrid was developed to take advantage of the strength of each component algorithm in performing feature selection. IG was used for primary selection because it was a simple and fast filter selection method; GA was used because it was a widely successful wrapper selection method that of which selection criteria included accurate classification performance, but it was used in a modified form because the conventional GA did not select a small enough number of features from a very large number of features; FFS was used because it was able to reduce the number of primarily selected features further based on the frequency of occurrences of a feature. In a previous study, the hybrid had successfully selected 142 most significant PCSNPs from a total of 16,579 SNPs in a smaller data set and provided a high classification accuracy [15].

#### Information gain

IG has been widely used in many machine learning tasks. It is well-known as a good filter method for text categorization task [17]. In recent years, IG has been improved to perform text categorization task more effectively [29, 30]. In a text categorization task, many irrelevant terms are mixed with a small number of significant terms in a collection of text documents in a similar manner to the presence of PCSNPs among many insignificant ones in a data set. Therefore, it was reasonable that we chose it to be a component of our developed hybrid. Moreover, IG has already been used for a similar kind of application to ours, such as gene selection in a gene expression task [5, 31]. In addition, IG has been successfully used in combination with SVM to classify cancer cases [32].

In the field of machine learning, IG, which is associated with informational entropy, is used to reduce the number of features. The IG value for a feature is the entropy of the whole data set minus the expected new entropy. The entropy in this sense is defined as the sum of the probability of occurrences of each class times the log probability of that class. If the features in a data set (a feature is an SNP in this study) are not very different, the entropy will be low and the IG value will be high. The features are ranked by their IG value in descending order. The top features are selected for use in the classification step. IG is calculated by (1) below,

1 $$  IG(T,X)=E(T)-\sum_{v\in Values(X)} \frac{|T_{v}|}{|T|}E(T_{v}),  $$

where *E*(*T*) is the entropy of the whole data in a training set calculated by (2) below,

2 $$  E(T)=-\sum_{i} p_{i}\log_{2}(p_{i}),  $$

where *T* is the training set; *X* is a feature; *T*_*v*_ is the subset of *T* for which feature *X* has a value *v*; *V* is all possible values of *X*; and *p*_*i*_ is the probability of class *i* computed as the proportion of class *i* in the training set.

#### Genetic algorithm

GA is a widely used wrapper method for feature selection [2, 4, 7, 33, 34] because the features selected from it are very efficient for a classification task in the field of ML. Nevertheless, in the case of a large number of features, the conventional GA cannot select a sufficiently small number of significant features [15]. Rathasamuth and her colleagues shows that the number of selected PCSNPs by GA was as high as a half of the whole set of the SNPs in the study, too high for porcine breed classification [15]. A suggestion has already been made that GA should be modified in order to achieve a small number of selected significant features [35]. In that study, GA was modified with a CHC algorithm. CHC algorithm employs a population elitist strategy, i.e., the best individuals of the following generation replace the worst individuals of the previous generation. Another feature of CHC is that even though individuals are selected randomly for recombination, they are allowed to mate only if their genomes are not very similar. CHC algorithm makes for more aggressive search. GA+CHC hybrid also finds an optimal solution faster than conventional GA. In the same vein, Li et al. reports a gene selection procedure by a GA-SVM hybrid on a set of microarray data, specially, the randomly-selecting-a-gene step in the GA procedure was modified to progressively reduce the number of genes to be selected by 50% in successive iterations [36]. In [8], the authors modified the mutation procedure of the original GA by assigning different values of the probability of bit-flipping from 0 to 1 and that from 1 to 0 in an attempt to reduce the number of selected features. In one of our previous studies [15], we employed this idea to modify GA that we subsequently used to successfully perform swine SNP selection. In this study, the prediction accuracy of SVM was used as the fitness function of GA, as shown in Algorithm 1.


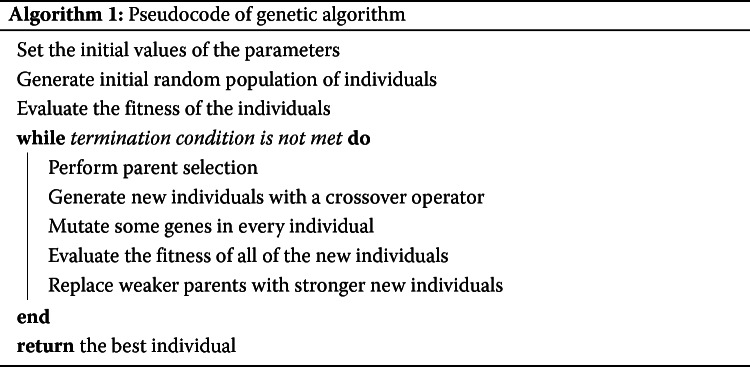


GA is an optimization algorithm based on random search. Basic components of GA include fitness function, chromosomes or individuals of the population, and genetic operators—selection (roulette wheel), crossover (multi-point), and mutation operators. GA mimics the processes of natural evolution and genetic heredity in finding an optimal solution. Each solution is represented by a chromosome which is recursively improved by genetic operators. In general, a solution or chromosome is represented by a string of binary numbers which is evaluated by a fitness function. A solution with the highest fitness value is the optimal solution.

GA attempts to find the best individuals in a population of individuals and have them reproduce better offspring. The offspring inherit the good characteristics of the parents and improve on those; therefore, they will have a good chance of survival. Algorithm 1 depicts the pseudocode of conventional GA. At the start, the initial values of various GA parameters are assigned: crossover rate, mutation rate, population size, and maximum number of generations. Next, an initial population of individuals is constructed. Each individual is represented by a string of binary numbers (0 and 1), and each binary number represents a gene or dimension of an individual or solution of the problem. Each individual is then evaluated of its fitness value by a fitness function. The probability that an individual will be selected to reproduce depends on its fitness value. In the parent selection step, individuals with high fitness values are selected to be crossed over together. This step is a significant step in GA that ensures that the search for the best solution is going in the right direction. The crossover positions of each pair of parents are randomly assigned. In order for a new solution to avoid getting trapped at a local optimum solution, some genes in the offspring will mutate with a random probability of bit-flipping mutation. After crossover and mutation, the fitness values of all of the offspring are evaluated and ranked together with the parent individuals. Next, the weaker parents are replaced by fitter offspring to be included in the next generation of population. The algorithm terminates when it reaches a maximum number of generations, and the fittest individual from the population is output as the optimal solution.

#### Support vector machine

SVM is a very effective classifier for problems with high-dimensional space. Applications of SVM are numerous such as text classification [37], classification in bioinformatics [1] including gene expression [31, 36], cancer [32, 35], and SNPs [9, 15]. SVM is a supervised learning classifier, i.e., it learns from a training data set. This model is further tested with a test data set. A properly trained model can classify whether an unknown sample is a member of which class. The idea behind SVM is to put the data into a feature space then determine the hyperplane with the highest margin that separates the data into two classes in that space. The data points used to construct the hyperplane are called support vectors. Originally, SVM was designed to be used with linear data; however, typical data are often non-linear, so kernel functions were brought in to deal with this issue. Kernel functions can be of many kinds. In this study, linear and radial basis function (RBF) kernels were used, and their performances were compared. A basic parameter for every SVM model is *C*, a hyperparameter that balances training error and model’s complexity. Another parameter especially for RBF kernel is *γ*, a kernel width. Both of these parameters need to be tuned properly in order to get an optimal hyperplane. The optimal values for these parameters can be obtained by a grid search. After properly tuned, these parameters are validated by a *k*-fold cross-validation procedure. The respective mathematical expressions for linear and RBF kernels are in (3) and (4),

3 $$\begin{array}{@{}rcl@{}} k(x,x') &=& x^{T}\cdot x',  \end{array} $$

4 $$\begin{array}{@{}rcl@{}} k(x,x') &=& \exp(-\gamma ||x-x'||^{2}), \end{array} $$

where *k*(*x*,*x*^′^) is a kernel on element *x* and *x*^′^ in the data set; ||(*x*−*x*^′^)||^2^ is the squared Euclidean distance between *x* and *x*^′^; and *γ* is a non-negative constant.

### Proposed methods

In previous studies, hybrids of IG and GA were used for feature selection [17, 38] and improving the precision of text categorization as well as reducing the high dimensionality of the text which could be as high as the number of swine SNPs used in this study. In our most recent study [15], IG+modified GA+FFS was successfully used to select PCSNPs which were then input into SVM to accurately classify swine breeds. FFS was used to select features with high frequency of occurrences in several randomly-seeded data sets derived from the training data set. The essential procedural steps of this method are described in the flowchart in Fig. 2. More detailed explanations about them can be found in [15]. Modified GA and FFS are explained in the immediate sections below followed later by an explanation of IG+modified GA+FFS.

#### Modified GA

The modified GA used in this study, the same one used in our most recent study [15], was modified from basic GA. In particular, the mutation operator was modified following the proposal in [8]. Basically, the modification assigns different mutation probabilities for flipping bits, from 1 to 0 versus from 0 to 1, in order to reduce the number of selected features. Bit flipping is done as expressed in (5) below,

5 $$  g(i)=\begin{cases}1 & ; r\le P_{m}\\0 & ;Otherwise\end{cases},  $$

where *g*(*i*) is the flipped bit at the position *i* of a mutating gene, *r* is a random number between 0 and 1, and *P*_*m*_ is the mutation rate.

#### Frequency feature selection

**Fig. 13:**
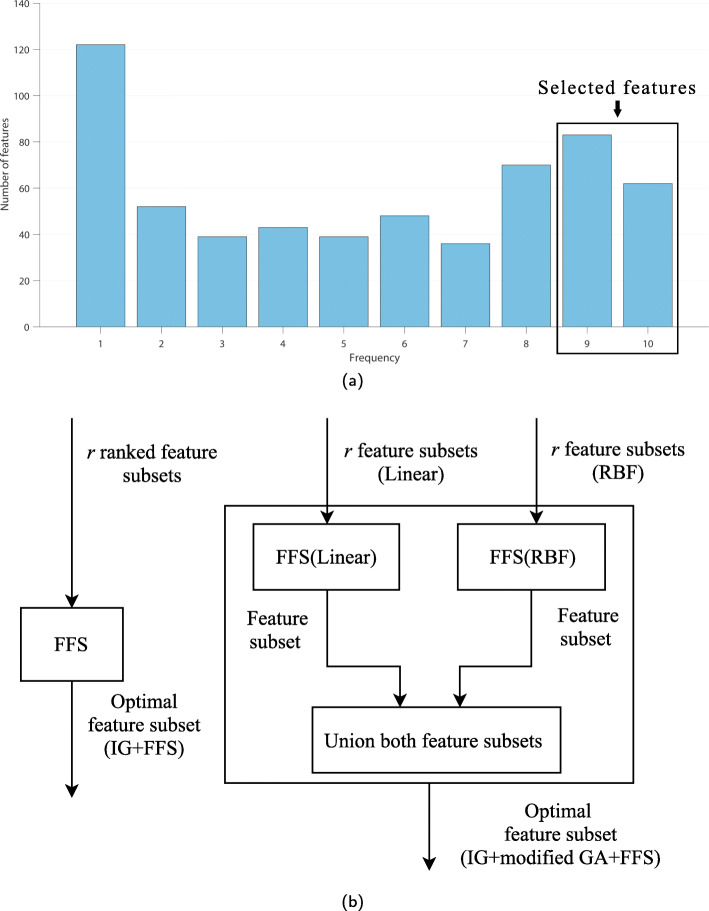
Frequency of occurrences of PCSNPs in all subsets of selected features from IG and IG+modified GA versus the number of PCSNPs that occurred with that frequency (**a**); and a schematic diagram of FFS operation (**b**)


Here, frequency feature selection means feature selection according to the frequency of occurrences of features that appear in every subset of features selected by IG and IG+modified GA. In our previous study [15], by using FFS, the number of PCSNPs selected by IG and IG+modified GA were reduced to a smaller number of the most significant ones. Moreover, IG+modified GA+FFS not only provided the smallest number of the best PCSNPs that provided the most accurate classification results. FFS procedure finds the frequency of occurrences of each feature in the entire randomly-seeded training data sets and selects only the features with equal or higher frequency of occurrences than a specified frequency threshold (*t*). For instance, if there are 10 randomly-seeded training sub-data sets and the frequency threshold for a feature was specified as 9, only the features that have the frequency of occurrences of 9 and 10 in all 10 randomly-seeded sub-data sets are selected. An example is shown in Fig. 13a. The higher the threshold, the smaller the number of features that get selected. In the previous study, the best value for this threshold, 8, was found from trial and error. In this study, since there were two feature subsets of features selected by IG+modified GA+FFS, one from the linear kernel and the other one from RBF kernel, we combined the selected features from both kernels to be the final subset of selected features, i.e., the final subset is the union of the selected features from both kernels. The reason for combining them was that the features selected by each kernel were high frequency features hence most relevant and significant, and combining them together should give us more classification power. FFS operation is shown in Fig. 13b, and the pseudocode of FFS is shown in Algorithm 2.

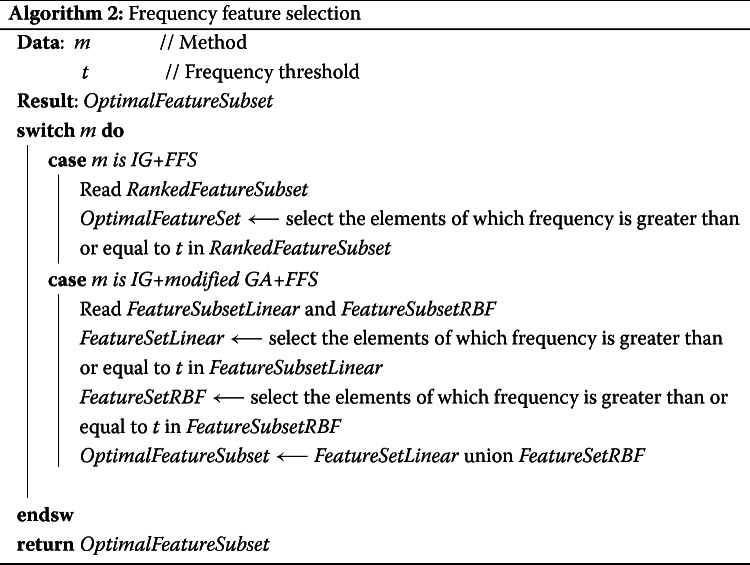


#### Hybrid of information gain, modified gA, and frequency feature selection with sVM

The pseudocode of IG+FFS and IG+modified GA+FFS in combination with SVM are shown in Algorithm 3. IG+FFS is a combination of a filter method and a selection method while IG+modified GA+FFS combines a filter method with a wrapper method. They had a distinct advantage of concise selection of statistically significant porcine-classifying SNPs (features), and, in particular, the FFS selection method contributed to more reduction of the number of selected features as demonstrated in [15].


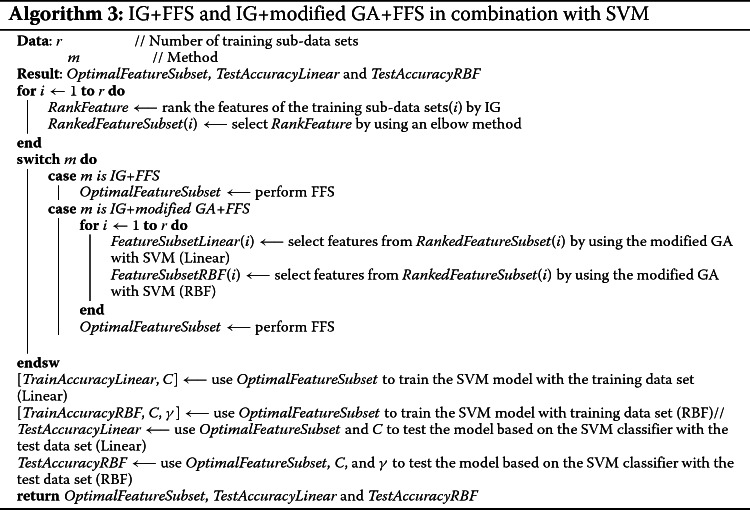


## Supplementary information

**Additional file 1** The selected PCSNPs from 10 randomly-seeded data sets. There are 341 selected PCSNPs by IG+modified GA+FFS from 10 randomly-seeded data sets.

## Data Availability

The data set generated and/or analysed during the current study is available in the GitHub repository, https://github.com/dsmlr/th-vn-us-swine. In addition, the selected PCSNPs from 10 randomly-seeded data sets are included in this published article’s supplementary information file.

## Abbreviations

Chr: Chromosome
FFS: Frequency feature selection
GA: Genetic algorithm
IG: Information gain
MAF: Minor allele frequency
PANTHER: Protein ANalysis through an evolutionary relationships
PCA: Principal component analysis
PCSNPs: Porcine-classifying single nucleotide polymorphisms
RBF: Radial basis function
SNP: Single nucleotide polymorphism
SVM: Support vector machine
